# Pelvic Peritonitis and Its Treatment

**Published:** 1894-05-26

**Authors:** James Oliver

**Affiliations:** Physician to the Hospital for Women, London


### Medical Progress and Hospital Clinics.

[*The Editor will be glad to receive offers of co-operation and contributions from members of the profession. All letters should be addressed to* The Editor, The Lodge, Porchester Square, London, W.]

### PELVIC PERITONITIS AND ITS TREATMENT.

By James Oliver, M.D., F.R.S.Edin., Physician to the Hospital for Women, London.

Pelvic peritonitis is an acute or chronic disease consisting in inflammation affecting, it may be, the whole, but more usually some part only, of that serous membrane which, located within the cavity of the pelvis, enters not only into the formation of its walls and floor, but covers more or less completely the organs contained therein. The expression includes, therefore, peri-metritis (inflammation of the covering of the womb), peri-oophritis (inflammation of the covering of the ovary), peri-salpingitis (inflammation of the covering of the Fallopian tube), and peri-proctitis (inflammation of the covering of the upper portion of the rectum).

The inflammatory disturbance, although originating locally in the pelvis, may nevertheless, in consequence of a direct continuity of tissue, spread to and affect the whole peritoneal sac.

Pelvic peritonitis is not only one of the most common but one of the most important of the diseases of women, for long after the subsidence of the disorder it may result in changes which tend to endanger life. Bands may constrict the Fallopian tube and favour the occurrence of tubal pregnancy by interrupting the impregnated ovum in its passage to the uterus, or the tube after the fimbriated extremity has become hermetically sealed may become distended with serum blood or pus.

Inflammation of the pelvic peritoneum may occur at any period of life. It most commonly develops, however, during the child-bearing epoch, and this, no doubt, is attributable to the physiological variations which may take place in the organs of generation during this period of life.

The inflammatory process may result in a thickening of the peritoneum and in adhesion of the opposing surfaces, and thereby the organs contained in the pelvis may be more or less firmly and completely glued together. The following is a typical case of this description, in which the disorder developed insidiously:—

D. D., æt. 25, and married five years, has never been pregnant. She began to menstruate at the age of 10. The discharge has usually lasted five days, but during the last two years it has gradually decreased in amount, and now it continues for two days only. During the four hours preceding the appearance of the menstrual discharge, as well as during the time that the flow continues, the patient complains of pain, which is sometimes referred to one iliac region and sometimes to the other. At the same time pain is experienced in the lower part of the back. Prior to two years ago the menstrual evolution occurred without producing either pain or discomfort.

Physical signs. The cervix is located towards the left side of the pelvis. The os looks downwards. Posteriorly in Douglas's pouch is felt the fundus uteri slightly flexed. On the left side is felt a small annulated swelling, which is rather fixed. On the right side is felt the ovary, which is enlarged and fixed.

On abdominal section the pelvic organs in this case were so extensively and firmly matted together that the ovaries and tubes as such could not be differentiated.

The material which exudes from the vessels during an attack of pelvic peritonitis may coagulate, and thus a tumour of greater or less size may be produced. The following is a fairly typical case of this variety.

Clara I——, æt. 25, and married five years, has had four children. The last child was born nine weeks before the patient came under my observation. For three weeks she has noticed a "lump" on the right side in the lower abdomen. Ever since the confinement she has complained of pain above the right groin. For three or four days after the confinement there was a very copious discharge of blood, and thereafter for fourteen days there was a greenish discharge. If the patient lies on her back she is more comfortable if the right leg is kept extended.

Physical signs. The abdomen, which is rather prominent on the right side, is occupied by a globular and regular swelling, which measures transversely $4\frac{1}{2}$ inches to the right and 1 inch to the left of the umbilical line. Its summit extends to $5\frac{3}{4}$ inches from the pubes. It is tender and appears to be solid.

The cervix uteri, which is located nearer the right than the left side of the pelvis, is somewhat closely applied to the pubes. The os which is patent looks downwards. On the right side of, and posteriorly to, the cervix, is felt a plastic exudation of a spleen-like consistence. This exudation is continuous with the body of the uterus and also with the abdominal mass already described.

The temperature was normal. In four weeks this exudation disappeared entirely, and three months later it was impossible to detect any trace of the pre-existing exudation.

By a process of fatty degeneration and liquifaction the plastic exudation may eventually become absorbed, and the rapidity with which complete resolution may be effected is sometimes very astonishing. Adhesions may, however, exist, and the uterus may consequently be more or less deviated and fixed.

In a few rare cases there is an effusion of serum, and this, when confined and large in amount, or rather when capable of being detected, constitutes what is termed encysted serous peritonitis. The following is a typical case of this description.

Ella R., æt. 20, and single, began to menstruate at the age of fourteen, and the flow has usually lasted seven days. Seven weeks ago, while menstruating, the patient began to complain of pain all over the lower abdomen, but more especially on the right side. Although usually unwell for seven days, on this occasion the menstrual discharge continued for fourteen days. For seven weeks she has complained of "fulness of the stomach," and of pain shooting down the right thigh as far as the knee. For six weeks she experienced pain during micturition, but during the last week the urine has been passed without pain. Temperature 101·4 deg. Fahr.

Physical signs, The abdomen is occupied by a somewhat pyriform swelling which reaches to the umbilicus. It is fairly central, but extends further to the right of the umbilical line than to the left. It is fluctuant. The cervix is slightly enlarged. To the right of the cervix is felt a softish body, which is fixed to the pelvic floor. The body of the uterus is incorporated with the abdominal swelling.

The abdominal swelling gradually disappeared, and after four months the following note was made. The abdominal tumour has entirely disappeared; the uterus as a whole is now closely applied to the right pelvic wall.

In this and another case of encysted serous peritonitis which has come under my observation, the fluid when it began to diminish was at first rapidly absorbed, as, however, it became more concentrated the process of absorption proceeded more slowly.

Suppuration and abscess formation is a not infrequent result of pelvic peritonitis. When this takes place the pus may escape externally by perforating the gut, the abdominal wall, the vagina, and the bladder, or, after burrowing in the tissues of the pelvis, it may escape from some opening in the perinæum. In a few rare cases the pus may make its way into the general cavity of the peritoneum, and so cause death.

In the majority of cases when suppuration occurs the pus escapes into the bowel, and the abscess is thus evacuated. In the following case the pus escaped in this manner:—

H. L., æt. 27, and single, began to menstruate at the age of eleven, and the discharge usually flows for three days. One month ago (after the patient had ceased menstruating three days) she experienced a sensation of chill whilst resting after a game at tennis. Almost immediately thereafter she complained of pain all over the lower abdomen, and simultaneously a hæmorrhagic discharge from the vagina was observed. The vaginal discharge persisted for three weeks, and occasionally clots were expelled. Four days after the cessation of the hæmorrhagic discharge I was asked to see the patient, as the abdominal pain was still great, and she was complaining much of cramp in the right leg. The temperature then was 100·2 deg. Fahr.

Physical signs. On the right side (iliac region) of the abdomen there is an ill-defined feeling of fulness, and the patient complains much of tenderness when this portion of the abdomen is palpated and percussed. The percussion note everywhere is resonant.

The cervix, which is fairly central, is pushed towards the pubes. Behind the cervix, and extending into the right fornix, is felt a somewhat globular swelling, which is incorporated with the pelvic floor, and which can be defined by bimanual examination. Its walls are thick, but the sensation is one of the presence of fluid.

Four days later a large quantity of pus was passed by the lower bowel. Thereafter the pelvic tumour was never again detected, and the patient rapidly recovered.

Two years later, when the patient was examined, as she complained of constant burning pain in the right groin, I found very decided puckering in Douglas's pouch, and in the right fornix of the vagina. The uterus was movable, but it was so much drawn to the right side that the fundus corresponded with the middle of the right Poupart ligament.

In pelvic peritonitis small suppurations frequently take place, but these, as distinct abscess formations, can seldom be detected by the most careful examination. Their probable existence in any given case is only confirmed when small quantities of pus are detected with the rectal evacuations.

I shall not here enter into detail regarding the causes of pelvic peritonitis. In many cases it is induced by the ill-advised use of injections, which are generally cold.

*Symptoms.*—Pain is the commonest symptom. In a large proportion of the cases vaginal hæmorrhage is observed, and in not a few the patient comes under observation on account of this discharge. It frequently happens that so long as the hæmorrhagic discharge flows from the vagina little or no pain is complained of. Pain either before or after passing water is a common accompaniment for a time of this disorder. It is not usually observed, however, until adhesions form. If the inflammatory exudation involves the sigmoid or upper portion of the rectum some discomfort or pain will, as a rule, be referred to the "back passage." The temperature of the body will vary in individual cases, and according to the time at which the patient is first seen. In many cases the disease develops so insidiously that it produces little or no constitutional disturbance. If suppuration occurs, then the temperature of the body reveals, as a rule, some indication of this variation.

*Treatment.*—In the more acute cases absolute rest should be enforced. The diet should be non-stimulating, and constipation should be avoided. If the pain is severe a few leeches may be applied to the lower abdomen, they are better applied here than to the cervix, as passing the speculum for the latter application often causes great pain, and may aggravate the disorder. Poultices may be applied, but the vaginal douche of plain hot water is, as a rule, more grateful and less risky. It may, so long as the patient is kept in bed, be used three or four times a day, and the temperature of the water should range from 105 to 120 deg. Fahr.

In acute cases salicylate of soda or phenazonum may be administered, or a mixture like the following will prove useful: ℞ Liq. bis. et am. cit., ʒj.; tr. cardamom co., ʒj.; liq. morphia acet., ♏x.; aq. ad., ℥j. every four or six hours. The administration of some lime salt from an early period is usually very efficacious, and a mixture like the following is that which I often employ: ℞ Calcii chlorid., grs. x.; glycerine, ʒs.; inf. calumb., ʒij.; aq. ad., ℥j. This should be taken three times a day after food, and if recommended, whilst the pain is still severe, small doses of liquor morphia hydrochloratis may be added to the above combination.

If suppuration occurs it may be necessary to open the abscess; if possible, however, nature should be guided, and allowed to determine the spot at which evacuation should take place.

In chronic cases counter-irritants may prove serviceable, such as the "fly blister," and iodine.

